# Learning from methylomes: epigenomic correlates of *Populus balsamifera* traits based on deep learning models of natural DNA methylation

**DOI:** 10.1111/pbi.13299

**Published:** 2019-12-18

**Authors:** Marc J. Champigny, Faride Unda, Oleksandr Skyba, Raju Y. Soolanayakanahally, Shawn D. Mansfield, Malcolm M. Campbell

**Affiliations:** ^1^ Department of Molecular and Cellular Biology University of Guelph Guelph ON Canada; ^2^ Department of Biological Sciences University of Toronto Scarborough Toronto ON Canada; ^3^ Department of Wood Science University of British Columbia Vancouver BC Canada; ^4^ Saskatoon Research and Development Centre Agriculture and Agri‐Food Canada Saskatoon SK Canada

**Keywords:** epigenomics, deep learning, authentication, poplar

## Abstract

Epigenomes have remarkable potential for the estimation of plant traits. This study tested the hypothesis that natural variation in DNA methylation can be used to estimate industrially important traits in a genetically diverse population of *Populus balsamifera* L. (balsam poplar) trees grown at two common garden sites. Statistical learning experiments enabled by deep learning models revealed that plant traits in novel genotypes can be modelled transparently using small numbers of methylated DNA predictors. Using this approach, tissue type, a nonheritable attribute, from which DNA methylomes were derived was assigned, and provenance, a purely heritable trait and an element of population structure, was determined. Significant proportions of phenotypic variance in quantitative wood traits, including total biomass (57.5%), wood density (40.9%), soluble lignin (25.3%) and cell wall carbohydrate (mannose: 44.8%) contents, were also explained from natural variation in DNA methylation. Modelling plant traits using DNA methylation can capture tissue‐specific epigenetic mechanisms underlying plant phenotypes in natural environments. DNA methylation‐based models offer new insight into natural epigenetic influence on plants and can be used as a strategy to validate the identity, provenance or quality of agroforestry products.

## Introduction

Modelling plant phenotypes using machine learning has significant potential to improve plant breeding and precision agriculture and is employed at several stages of the agricultural cycle. Supervised statistical learning, a form of machine learning, comprises a suite of modelling techniques that learn generalizable patterns within examples of training data. Genomic prediction is an example of supervised statistical learning that uses a variety of machine learning methods to model associations between genotype and phenotype (Libbrecht and Noble, 2015). Models of this kind allow breeders to focus attention on new varieties predicted to exhibit desirable traits (Libbrecht and Noble, 2015; Yu *et al.*, 2016). Although few genomic prediction studies have been reported using tree species, growth trait models in *Eucalyptus* explained significant proportions of heritable variance with a small number of significant associations detected by GWAS (Müller *et al.*, 2017).

In addition to genomic prediction of future agricultural performance, machine learning is applied to photographs or spectral imagery captured by remote sensing technologies in order to diagnose the health and productivity of crops. Computer vision models find patterns in spatially organized data which are reliable estimators of soil composition, abiotic stress and plant disease (Ghosal *et al.*, 2018; Perez‐Sanz *et al.*, 2017). Machine learning finds equal application at the end of the agricultural cycle in the form of methods to authenticate the identity or provenance of agroforestry products (Zhao *et al.*, 2014). Whether based on stable isotope ratios or patterns of abundance of specific metabolites, fingerprinting of these variables is a developing technology to validate where food products originate or deduce conditions under which agricultural materials are produced (Li *et al.*, 2016; Cubero‐Leon *et al*., 2018).

As with crop species, recent research in silviculture has employed statistical learning to predict industrially important traits such as total biomass and wood density in trees. Metabolic fingerprinting is capable of discriminating among hybrid poplar (*Populus tremula* × *alba*) genotypes exhibiting differences in wood traits (Robinson *et al.*, 2005). Bayesian learning networks incorporating transcript abundance and SNP‐level genotyping are effective classifiers of wood properties in *P. trichocarpa* (Porth et al., 2013a,2013b). Neural networks demonstrate nonlinear relationships between mechanical wood properties and other attributes such as tree height, diameter and acoustic velocity in Douglas fir (*Pseudotsuga menziesii* (Mirb.) Franco var. *menziesi*) (Iliadis *et al.*, 2013).

Cytosine methylation, a source of genome‐wide epigenetic variation, is a promising multipurpose estimator of plant traits. Analysis of near‐isogenic *Arabidopsis thaliana* lines segregating for DNA methylation differences showed that phenotypic variance in several heritable traits such as flowering time, primary root length and height is, to a significant degree, explained by variance in cytosine methylation (Cortijo *et al.*, 2014; Hu *et al.*, 2015; Kooke *et al.*, 2015). These studies provide evidence for novel mechanisms of evolutionary change, namely that heritable changes in DNA methylation may be targets for natural selection (Cortijo *et al.*, 2014; Dublin *et al.*, 2015).

Although the aforementioned laboratory experiments were conducted with mutants to induce methylation differences (Hirochika *et al.*, 2000; Jeddeloh *et al.*, 1998; Kakatuni *et al.*, 1999), they also raise the possibility of using natural epigenetic variation to model plant traits. Phenotypic estimation using epigenetic variation may be advantageous, as natural cytosine methylation is reversible and responsive to changes in genotype, tissue type and environmental stimuli (Meyer, 2011). Patterns of DNA methylation could be used to estimate industrially important traits in natural environments. To the extent plant traits correlate with DNA methylation, epigenome‐based models have significant potential in authenticating agroforestry products with respect to identity, provenance or quality.

Deep learning models are artificial neural networks in which nodes arranged in multiple hidden layers learn representations of the input data at different levels of abstraction (LeCun *et al.*, 2015). These models have achieved superior performance on statistical learning tasks and have been applied successfully in several areas of genomics, such as identifying splice sites associated with human disease (Leung *et al.*, 2014; Xiong *et al.*, 2015; Alipinahi et al., 2015) and predicting patient–drug interactions (Angermueller *et al.*, 2016; Yu *et al*., 2016).

This report tests the hypothesis that plant traits can be modelled from patterns of natural variation in genome‐wide cytosine methylation. Estimations of a purely genetic trait (provenance), a nonheritable attribute (tissue type) and weakly heritable physico‐chemical wood quality traits (biomass, density, mannose and soluble lignin contents) were performed on xylem and leaf DNA methylomes derived from genetically diverse balsam poplar trees grown at two common garden sites in Saskatchewan, Canada (Bräutigam *et al.*, 2017). The motivation to model associations using deep learning was driven by findings in genomics and other disciplines showing that artificial neural networks can be interpreted in detail (LeCun *et al.*, 2015). In the study described here, feed‐forward neural networks with two hidden layers were used to model balsam poplar traits based on selections of individual cytosine (CpG) methylation intensities as the input layer.

## Results and discussion

### Phenotyping and bisulphite sequencing of natural Populus balsamifera populations grown in two common gardens

**Figure 1:**
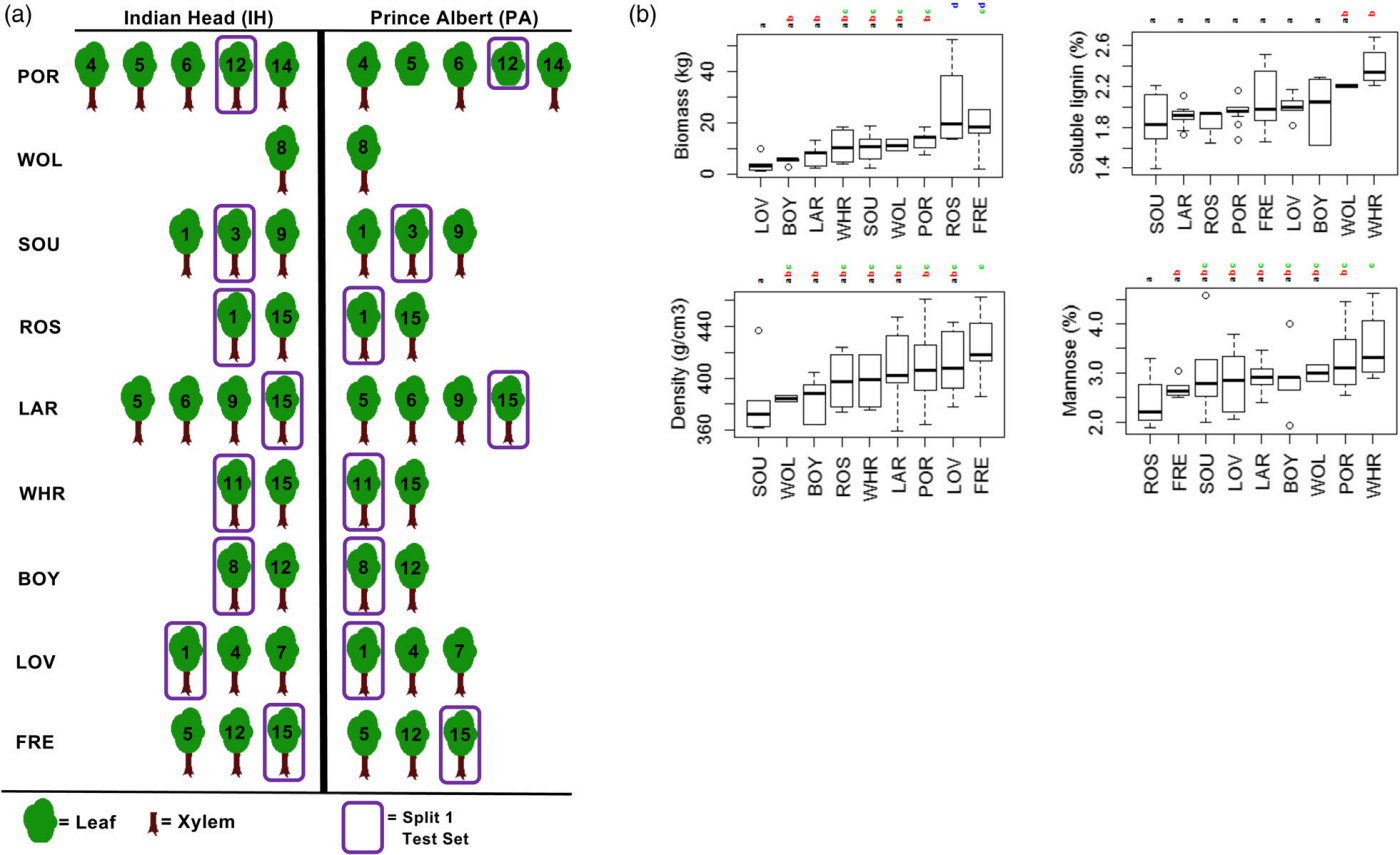
Study design and phenotyping of natural *Populus balsamifera* populations grown in two common gardens. (a) Schematic illustration of the study design. Twenty‐five *P. balsamifera* genotypes were replicated between two field sites, Indian Head and Prince Albert, Saskatchewan, Canada. Three‐letter code indicates the provenance of the trees from which germplasm was sampled (see Methods). The number assigned to each tree represents a distinct genotype within a provenance. Genotypes in purple boxes comprise the test set on which phenotypic predictions were evaluated. (b) Box plots of tree biomass, wood density, soluble lignin content and mannose content after 6 years of growth. Trees are grouped by provenance, and letters indicate significant differences among groups according to Tukey’s HSD test.

The *P. balsamifera* genotypes analysed constitute a subset of a larger Agriculture Canada Balsam Poplar (AgCanBaP) collection (Soolanayakanahally *et al.*, 2009). Dormant whips were harvested from *P. balsamifera* individuals in 9 natural populations located across Canada; thus, the location or population of origin represents the provenance of the trees (see Methods). Clones derived from *P. balsamifera* provenances were then replicated between two common garden sites established in Prince Albert and Indian Head, Saskatchewan, and grown for 6 years (Figure 1a).

Trees in the AgCanBaP collection were selected for modelling in this study based on evidence of natural genetic and environmental influence on their wood traits. As such, there were significant differences in biomass and wood properties among the provenances (Figure 1b) as well as differences in tree growth between the two common garden sites. As examples, trees of the Sioux Lookout (SOU), Roseville (ROS) and La Ronge (LAR) provenances grew significantly larger in Indian Head, whereas trees of the Whitehorse (WHR) and Boyle (BOY) provenances were larger in Prince Albert (Table S1).

Trees were harvested after leaf drop in 2011 and phenotyped (McKown et al., 2014; Porth et al., 2013a,2013b) with respect to their biomass and wood quality characteristics including density, soluble lignin and mannose contents (Figure 1b). During the summer of 2011, xylem tissue samples were collected from each poplar genotype grown at both locations. In the summer of 2014, fully expanded leaves from the same coppiced trees were collected in the same common gardens as 2011.

To obtain quantitative, genome‐wide variation in DNA methylation among the trees, whole‐genome bisulphite sequencing (Lister *et al.*, 2008) was conducted on 105 leaf and xylem tissue samples (Figure S1). The xylem DNA methylomes are identical to those reported previously on the epigenomic determinants of gender in balsam poplar (Bräutigam *et al.*, 2017). In this study, cytosine methylation in the CpG context was used exclusively as a quantitative predictor (see Experimental Procedures). CpG covered by> 10 sequences per methylome was filter selected. Consequently, the methylation data used for modelling consisted of 12,358,704 CpG methylation intensities per sample.

**Figure 2:**
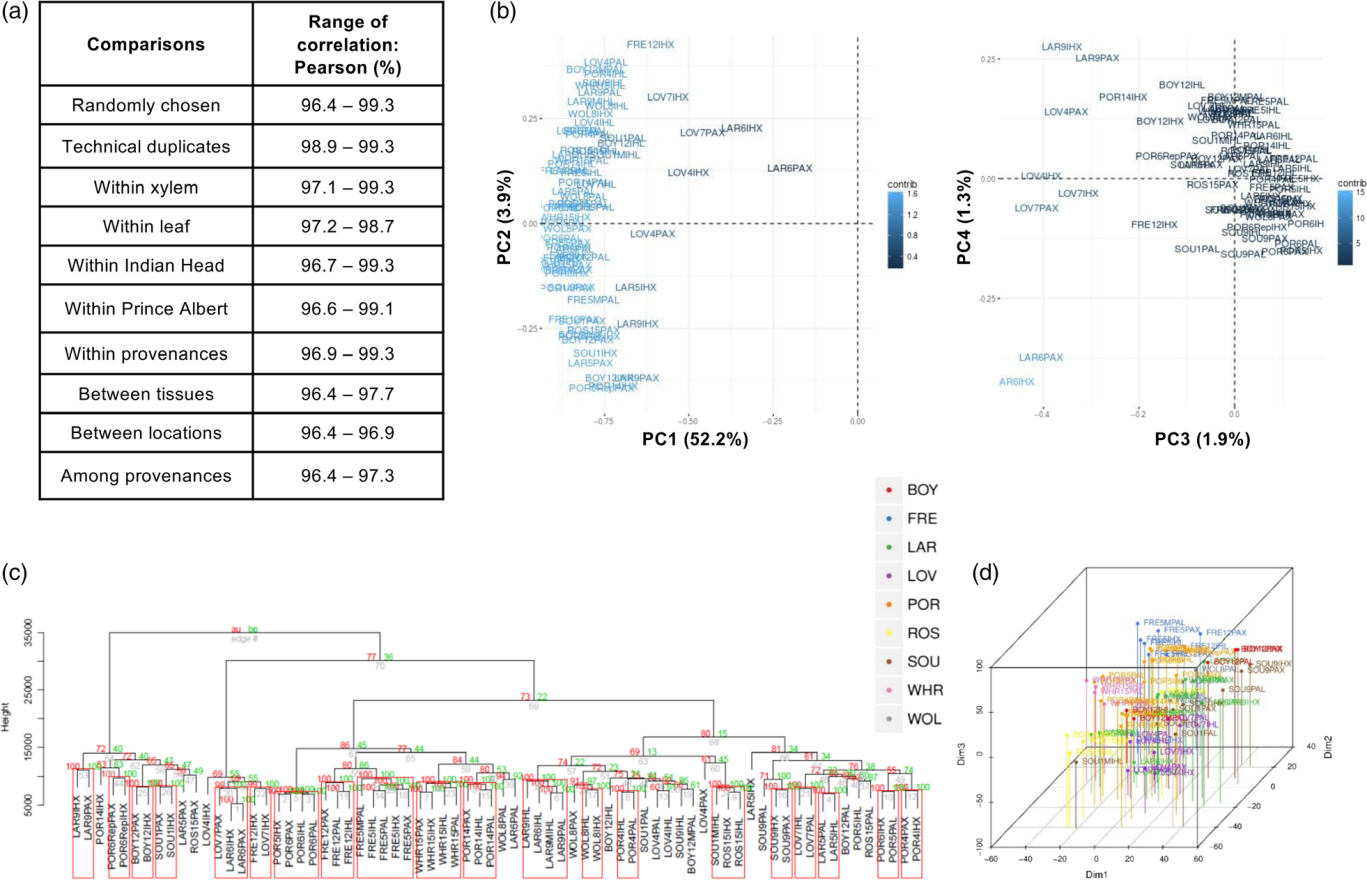
Exploratory analyses of CpG methylation in the training set. Genome‐wide patterns of CpG methylation in 72 methylomes comprising the training set. (a) Pearson’s correlation coefficient calculated on each pairwise combination of methylomes, using the set of 12358704 CpG covered by sequencing. (b) PCA conducted using this set of CpG. Distribution of methylomes is presented along PC1 and PC2 axes (left) and along PC3 and PC4 axes (right). Colour scale indicates contribution of methylomes to principal components. (c) Hierarchical clustering conducted on 200,000 randomly selected CpG using the Ward clustering method on Euclidean distances. Rectangles surround clusters supported at 0.95 level of significance. *p*‐Values denoted in green are estimated as bootstrap probabilities (bp) calculated using 1,000 bootstrap iterations. *p*‐Values denoted in red are approximately unbiased (au). (d) t‐SNE representation in 3 dimensions, calculated with the same 200,000 CpG used in (c).

The 105 CpG methylomes were divided into training and testing sets for statistical learning. With the exception of the Whitehorse (WHR) provenance, methylomes derived from one randomly selected genotype of each provenance were used as the test set (boxes in Figure 1a). This experimental design enabled examination of the flexibility of CpG methylation as a trait predictor, as methylomes of the same genotype varied according to tissue differences, the location of the trees and a time interval of three years (Figure 2).

### Exploratory analyses of genome‐wide CpG methylation in diverse balsam poplar methylomes

Methylomes were individually named according to genotype, tissue type and their location (Table S1). The first three letters (WHR, POR, etc.) designate the provenance of the trees, and the subsequent number designates a specific genotype within a provenance. The location of the trees in one of the two common gardens is given by IH or PA, and the tissue from which the methylomes were derived is given by X or L. For a small number of methylomes, tissue was collected from leaves at a more mature stage of development (designated ‘M’), and two methylomes were generated as technical duplicates using an identical tissue sample (designated ‘Rep’).

Pairwise comparisons showed that methylomes were highly correlated (96.4%–99.3%, Figure 2a). To examine global patterns of CpG methylation in the poplar methylomes, PCA, hierarchical clustering and t‐SNE (van der Maaten and Hinton, 2008) in three dimensions were performed on the 72 methylomes comprising the training data. PC1 accounted for 52.2% of variance in the methylation data and likely explains variance in genome‐wide average levels of CpG methylation. The LOV‐ and LAR‐derived methylomes separated from the main cluster along the PC1 axis are characterized by the lowest genome‐wide levels of CpG methylation (Figure S1g). With the exception of this group of methylomes, tissue of origin was separated along the PC2 axis (Figure 2b). Methylomes derived from the FRE (southern) and WHR (northern) provenances grouped into distinct clusters using HCA (Figure 2c) and were discriminated into groups by t‐SNE (Figure 2d), indicating underlying patterning in the methylation data with respect to provenance. The patterns observed are consistent with the high degree of dimensionality inherent in the experiments; therefore, further work focused on differential methylation as a biologically intuitive method to select cytosines for modelling.

### Epigenomic estimation of tissue type, a nonheritable trait

**Figure 3:**
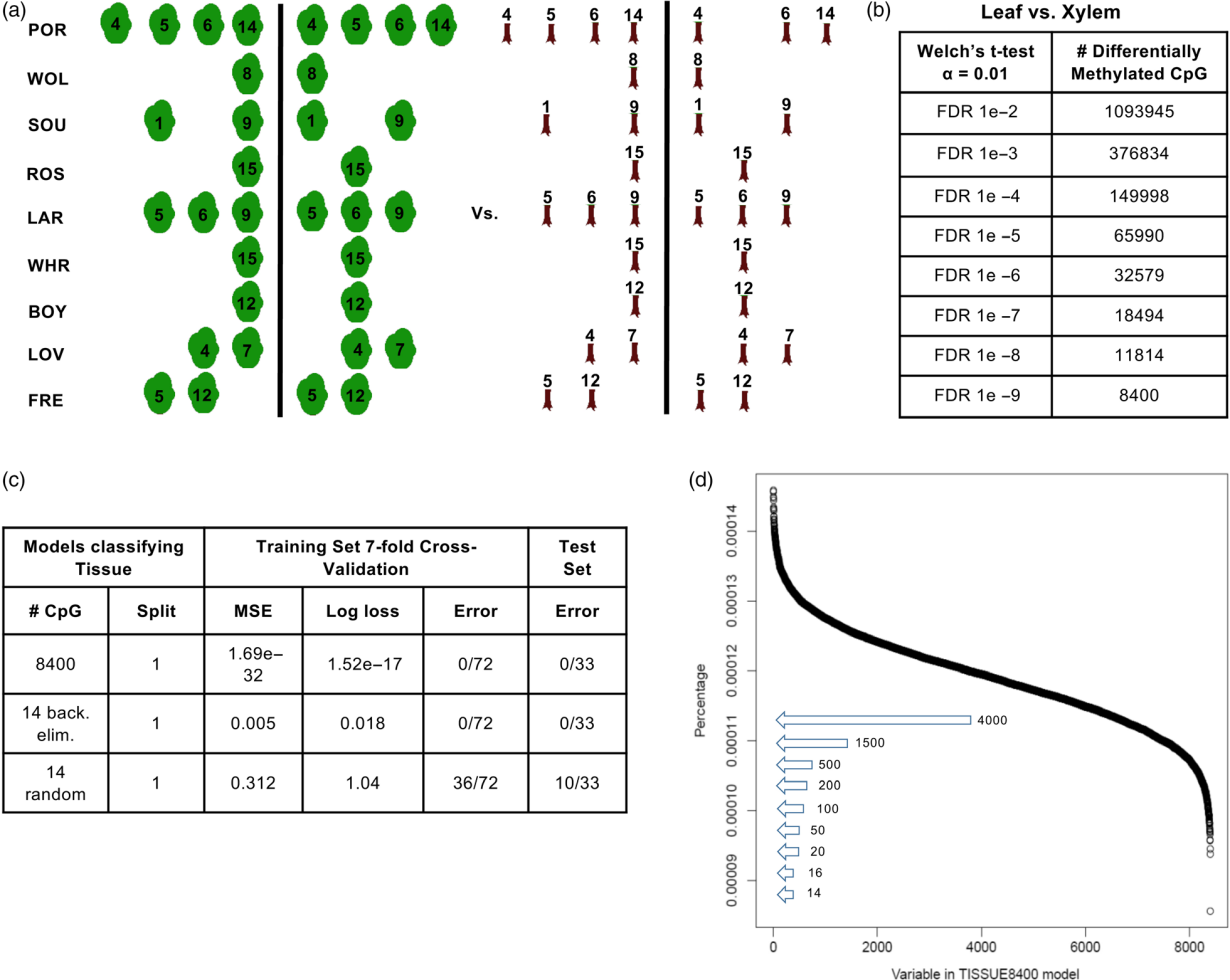
Variable selection and deep learning models classifying tissue type. (a) Schematic illustration of the differential methylation strategy used on 72 methylomes comprising the training set. (b) Differential methylation calculated using Welch’s *t*‐test on the training set at *α* = 0.01 level of significance and FDR at increasing levels of stringency. (c) Summary statistics of model fit and performance after sevenfold cross‐validation of the training set and on test set predictions. Cytosines used in modelling were either 8,400 CpG selected by differential methylation, 14 CpG selected by further backward elimination or 14 CpG randomly selected. MSE denotes mean squared error, and error denotes the misclassification rate. (d) Variable selection by backward elimination. Each of the 8,400 CpG used in the initial tissue prediction model is plotted according to its variable importance (open circles). Arrows indicate the number of CpG selected for modelling according to variable importance calculated in successively smaller models.

Tissue‐specific methylation in black cottonwood (*P. trichocarpa* Torr. & A. Gray) can be visualized on a chromosomal scale (Vining *et al.*, 2012). We hypothesized that tissues (xylem and leaf) could be discriminated in a quantitative manner using methylation data selected from a pool of differentially methylated cytosines. Using the full 12.5 million CpG covered by sequencing, a Welch’s t‐test was employed in a differential methylation strategy to compare all leaf‐derived methylomes with all xylem‐derived methylomes in the training set (Figure 3a). A total of 8400 high‐confidence CpG were identified at *α* = 0.01 with FDR 1e‐9 (Figure 3b).

Using the 8400 methylation intensities as the input layer, fast‐forward neural networks were trained to perform a binary classification of the tissue source of methylomes in the training set. Hyperparameter tuning searches were used to assess different setting combinations of the tuning parameters, including the number of neurons in hidden layers and the activation function used (Table S2a). Given that the number of training examples is small, random dropout (Srivastiva et al., 2014) and L1/L2 regularization (Bengio, 1998) techniques were employed at each layer to minimize overfitting.

Models trained in hyperparameter searches were then evaluated based on minimization of log loss (cross‐entropy) after sevenfold cross‐validation of the training data. The best‐fit tissue model resulted in a log loss of 1.52e‐17, and cross‐validation predictions correctly classified 72/72 members of the training set (Figure 3c). The fit model also classified the tissue source of 33/33 methylomes derived from novel genotypes of the test set (Figure 3c). To further examine a reliability estimate of this model, probabilities associated with each test set classification were calculated as the relative activation of the two neurons in the output layer. The probabilities of misclassification in the test set were much lower than expected, reaching a maximum of 3.39e‐28 for the POR12IHX methylome (Table S3a).

These results demonstrate that tissue type can indeed be classified with extremely high probability based on patterns of tissue‐specific DNA methylation. We hypothesized that simpler models could be successfully trained using a subset of the 8400 CpG. A backward elimination approach was therefore used to exclude CpG based on ranking the relative variable importance of each CpG in the input layer (Gedeon, 1997). Successively smaller models were trained using hyperparameter searches complemented with variable importance computations at 8400, 4000, 1500, 500, 200, 100, 50, 20, 16, 14 and 12 CpG as input (Figure 3d, Table S2a).

The best‐fit model obtained using 14 CpG as input had a log loss of 0.018, and cross‐validation predictions correctly classified the tissue source of all methylomes in the training set (Figure 3c). The backward elimination model also correctly classified the tissue source of all methylomes in the test set. The maximum probability of misclassification associated with novel genotypes was 0.87% observed for the ROS1PAL methylome (Table S3b).

Taken together, results demonstrated that classification and regression neural networks (see below) can be used to model several traits with small numbers of CpG predictors. Therefore, visual interpretations of the DNA methylation models were developed to examine how few CpG participate in accurate trait estimations. Features used by deep neural networks to make predictions can be quite transparent, and the method used here is similar to an approach used to identify DNA splice site mutations associated with disease (Alipahani et al., 2015).

**Figure 4:**
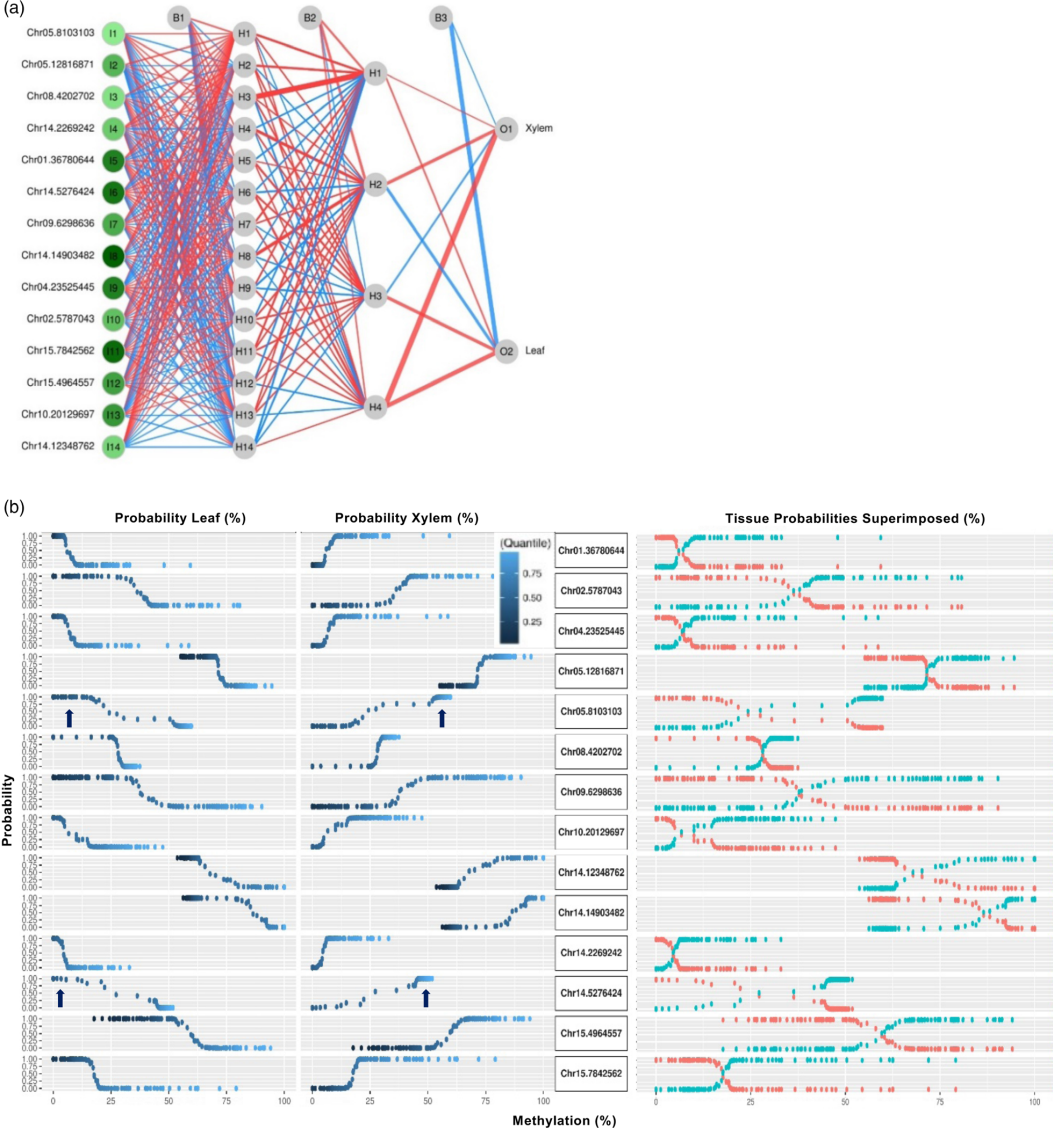
Interpretation of a model classifying tissue based on methylation of 14 CpG. (a) Neural interpretation diagram. I—input neuron, O—output neuron, H—hidden neuron, B—bias neuron. Red—stimulatory connection, blue—inhibitory connection. Line width is proportional to the magnitude of the connection. Input neurons are shaded from bright to dark green according to diminishing variable importance. (b) Methylation‐response plots illustrating the relationship between cytosine methylation level and the probability associated with each tissue. For each of the 14 CpG, points are plotted separately by tissue class (left), and for both tissue classes together (right).

The connectivity of the 14 CpG tissue model was examined using a neural interpretation diagram, in which the magnitude of the variable importance levels and neural weights and biases is illustrated (Figure 4a). The network weights and biases were then used to probe relationships between input variables and the output tissue estimations (Gevrey *et al.*, 2003; Lek *et al.*, 1996). A data permutation approach (see Methods) was employed to generate 137,214 different combinations of methylation values for the 14 input CpG. Using the learned network weights and biases, predictions were made for each of these permutations and tissue probabilities were plotted as a function of methylation level. The resulting ‘methylation‐response plots’ for each input CpG revealed striking nonlinear relationships between levels of methylation and the two tissue types (Figure 4b, Figure S2a).

For every CpG in the model, probabilities associated with leaf classification peaked at a *lower* level of methylation relative to xylem classification (Figure 4b—right). As such, methylation levels associated with tissue predictions corresponded to features found in the majority of the training methylomes (Figure S4). For two of the CpG, predictions seemed to identify features found in a minority of methylomes. Clustering (Figure S4) and differential methylation (Table S4) show the leaf‐derived methylomes were, on average, significantly more methylated at CpG located at Chr05.8103103 and Chr14.5276424 (compare arrows in Figure 4b with asterisks in Figure S4). Intervals associated with nearly 100% probability correspond to effective classifying features because they also closely match methylation levels found in novel genotypes (Figure S4).

As controls, deep learning models were fit to the training methylomes using three groups of 14 CpG randomly selected from the full set of 12.5 million. The best model obtained from hyperparameter searches using these randomly chosen CpG misclassified 36/72 methylomes in the cross‐validated training data and misclassified 10/33 of the test methylomes (Figure 3c). Relationships between input methylation and the output predictions could not be identified using methylation‐response plots calculated from these poorly performing models (Figure S2c).

An initial hypothesis was that the highly accurate methylation model would yield insight into epigenetic regulation of genes known to be differentially expressed between xylem and leaves, such as those involved in photosynthesis or lignification. However, the genomic context of tissue‐classifying CpG as well as methylation‐response plots suggests a different biological interpretation. Methylation‐response plots show that the model is attuned to specific intervals of methylation. To examine how the model might estimate absolute methylation levels in the two tissues, the 14 tissue‐classifying CpG were annotated and found to be distributed among nine different chromosomes at 14 distinct loci (Table S4). Two genes, *MMT* and *CMT2*, shared highest sequence similarity to known methyltransferases in *Arabidopsis*. *Potri.004G229800* (*MMT*) is proposed to encode a methionine S‐methyltransferase that regulates the available pool of methyl groups by catalysing the synthesis of S‐methyl methionine from S‐adenosyl methionine (Kocsis *et al.*, 2003). *Potri.014G179900* (*CMT2*) is proposed to encode a cytosine‐5‐methyltransferase, catalysing the methylation of DNA using S‐adenosyl methionine as a methyl donor (Bartee *et al.*, 2001; Stroud *et al.*, 2013). CMT methyltransferases have been identified only in plants and are involved in establishing global differences in DNA methylation observed during development and adaptation to local environments (Shen *et al.*, 2014; Stroud *et al.*, 2014) Regulation of *CMT2* and *MMT* orthologues may point to an epigenetic mechanism underlying tissue discrimination in balsam poplar.

### Estimation of provenance, an element of population structure

**Figure 5:**
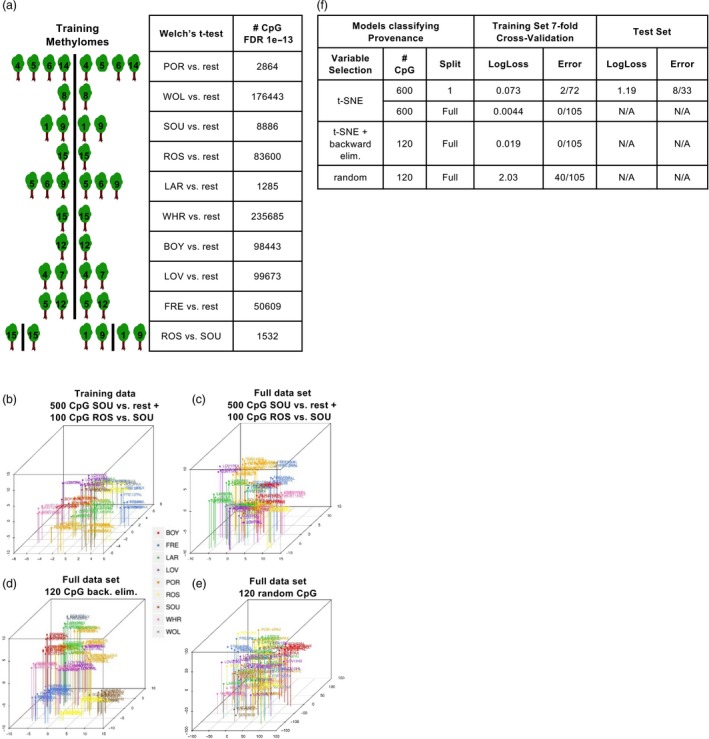
Variable selection and deep learning models classifying poplar provenances. (a) Schematic of differential methylation tests used on methylomes comprising the training set. Each provenance was compared with the remaining provenances, and, at the bottom, SOU‐derived methylomes were compared with ROS‐derived methylomes. (b) t‐SNE of methylomes in the training set computed with the indicated groups of cytosines. (c, d, e) t‐SNE of methylomes in the full data set computed with the indicated groups of cytosines. (f) Summary statistics of models classifying provenance. Cytosines used in modelling were 600 CpG selected from the differentially methylated cytosines by t‐SNE visualizations, 120 CpG selected by further backward elimination of cytosines or a random selection of 120 CpG.

t‐SNE analyses of genome‐wide CpG methylation in the training data showed partial clustering of genotypes into their respective provenances (Figure 2d). A more complete separation of provenances might be achieved by selecting and visualizing combinations of differentially methylated CpG. As such, a Welch’s *t*‐test was used to identify CpG differentially methylated using each provenance in comparison with the remaining eight (Figure 5a) and between two provenances in all possible pairwise combinations of the training data (only ROS vs. SOU is shown in Figure 5a).

t‐SNE conducted with as few as 600 differentially methylated CpG showed that provenances could indeed be well separated (compare Figure 5b with Figure 2d). The initial set of models built to classify provenance were therefore based on a particular pooling of 600 CpG: 500 CpG derived from the SOU vs. rest differential methylation test and an additional 100 CpG derived from ROS vs. SOU (Figure 5b).

With this set of 600 CpG as the methylation input layer, models were trained to classify the provenance of the training methylomes (Table S2b). The best‐fit provenance model misclassified only 2/72 methylomes in the cross‐validated training data (Figure 5f). However, 7/33 methylomes comprising the test set were incorrectly classified, notably all four methylomes derived from the ROS1 genotype (Figure 5f, Figure S3a). Confusion matrices obtained in this hyperparameter search showed the four ROS1‐derived methylomes were incorrectly classified in 97% of models.

Provenance classification results suggested the ROS1 genotype could be an outlier with respect to its epigenotype. Therefore, admixture analysis was conducted using each *P. balsamifera* genotype as well as common *P. trichocarpa* and *P. deltoides* genotypes. All trees of the ROS provenance (ROS1 and ROS15 in this study) proved to actually be *P. balsamifera × P. deltoides* hybrids (Bräutigam *et al.*, 2017). Given this important distinction between the ROS trees and the other provenances, it is unsurprising that provenance estimations of ROS‐derived samples were poor in the test set.

Better‐performing provenance models might be obtained by including all methylomes (with the 8 ROS‐derived methylomes) as training examples. Consequently, identical differential methylation tests and visualizations selecting 600 CpG were conducted using the total set of methylomes (Figure 5c). The best‐fit provenance model constructed using the full set of 105 methylomes was characterized by a sharp decrease in log loss (0.0044) and cross‐validation results correctly classified every member of the complete data set (Figure 5f, Figure S3b). Probabilities associated with each provenance classification exceeded 99.9% (Table S3c). Therefore, provenance was classified with high probability using 600 CpG and 105 methylomes as training examples, but performance markedly decreased when only 72 methylomes were used for variable selection and model training.

Successively smaller provenance models were trained using variable importance computations eliminating 5% of the 600 input CpG at each step. The smallest model found classifying all methylomes of the cross‐validated data set used 120 CpG methylation intensities as an input (Figure 5d,f). As comparators, we attempted to classify provenance using three groups of 120 CpG randomly chosen from the full set covered by sequencing (Figure 5e). Based on these random selections, the best predictions misclassified 40/105 methylomes (Figure 5f, Figure S3b).

**Figure 6:**
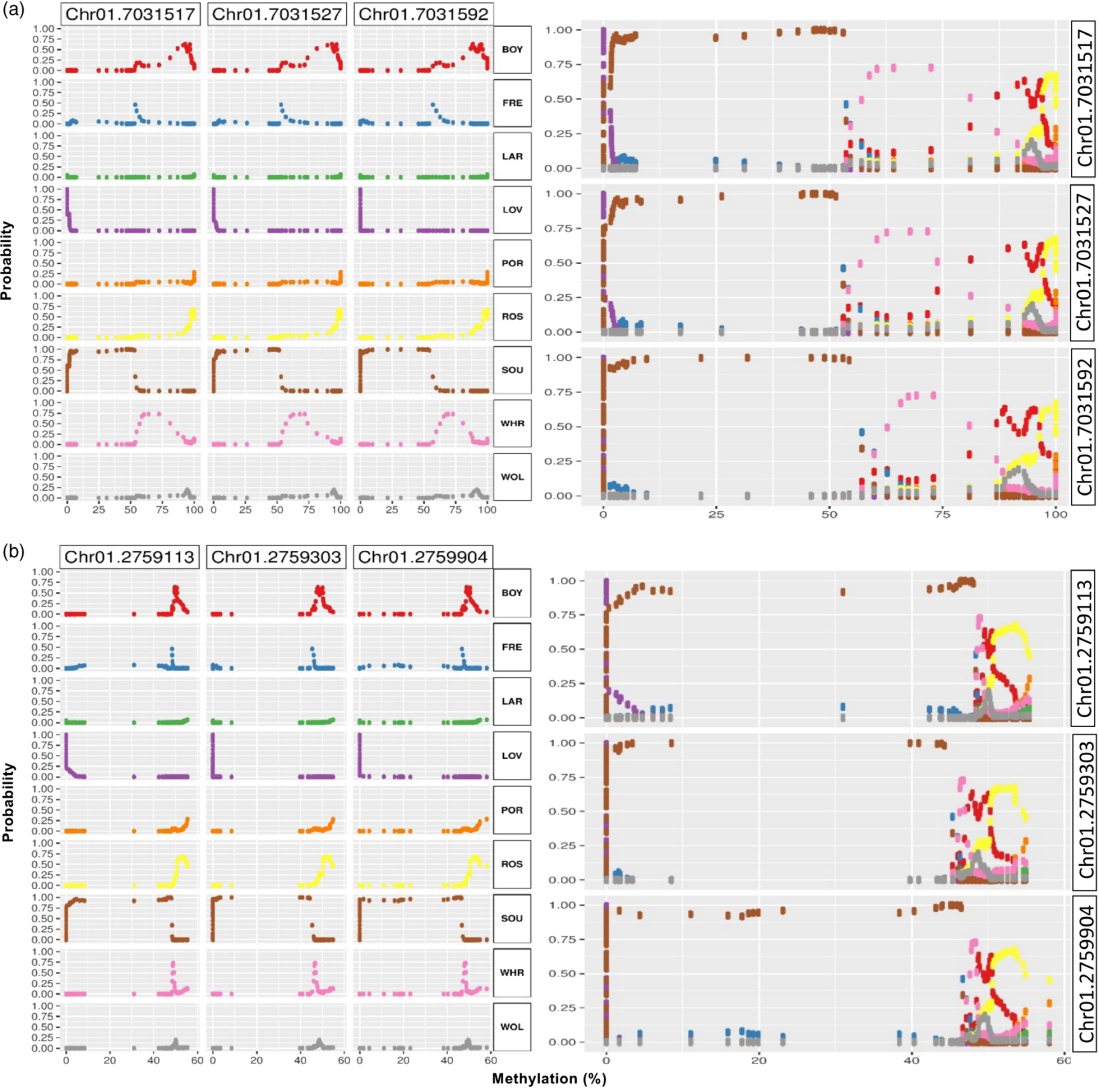
Interpretation of models classifying provenance based on methylation of 120 CpG. Methylation‐response plots illustrating the relationship between cytosine methylation and the probability associated with each provenance. For the indicated CpG, points are plotted separately for each provenance (left) and for all provenances together (right). (a) Methylation‐response plots calculated from the model presented in Figure 5d, f. Curves are shown for three CpG residing in the Potri.001G088900 locus. (b) Methylation‐response plots calculated for three CpG residing 50 kb upstream of the Potri.001G038700 locus in the same best‐fit deep learning model.

Methylation‐response plots for three CpG residing in the Potri.001G088900 locus on chromosome 1 (encoding a putative glutaredoxin family protein) were generated from 1.2 million permutations using the best performing 120 CpG provenance model (Figure 6a). For each of the three CpG predictors, maximum probabilities for class predictions can be identified in a sequence from lowest to highest level of methylation: LOV, SOU, FRE, WHR, BOY, WOL, ROS, LAR and POR. The sequence in which probabilities peaked was not dependent on locus or relative variable importance and was invariant for every CpG in the model. To further demonstrate this, methylation‐response curves are shown for three distinct CpG, located in an intergenic region approximately 50 kb upstream of the Potri.001G038700 locus on chromosome 1 (Figure 6b). Specific levels of methylation associated with maximum probabilities are different between the two regions, but peaks occur in an identical sequence. In this model, particularly large intervals of methylation distinguish the SOU‐derived methylomes from the other provenances (Figure 6), which is reflective of the differential methylation strategy used to select CpG.

The invariant sequence of peaks relating methylation levels to output probabilities reveals a pattern used by deep learning to perform classifications with very few CpG. Classification was accomplished based on the insight that there is an optimal ordering or sequence of classes with respect to methylation level. Methylation‐response visualizations thus appear to show the learned intervals or levels of methylation for each CpG used to distinguish the provenances in precisely the same sequence (Figure 6). Poorly performing provenance model built using 120 randomly selected CpG as input might use a different computation compared to well‐fit models. Methylation‐response plots showed that the ordering of classes with respect to methylation differences was, in fact, different in comparison with highly predictive models (Figure S3d). This mechanism also explains the unusual way in which tissue type can be classified by associating xylem with a higher level of methylation than leaves (Figure S4b).

### Tissue‐independent epigenomic estimation of tree biomass

**Figure 7:**
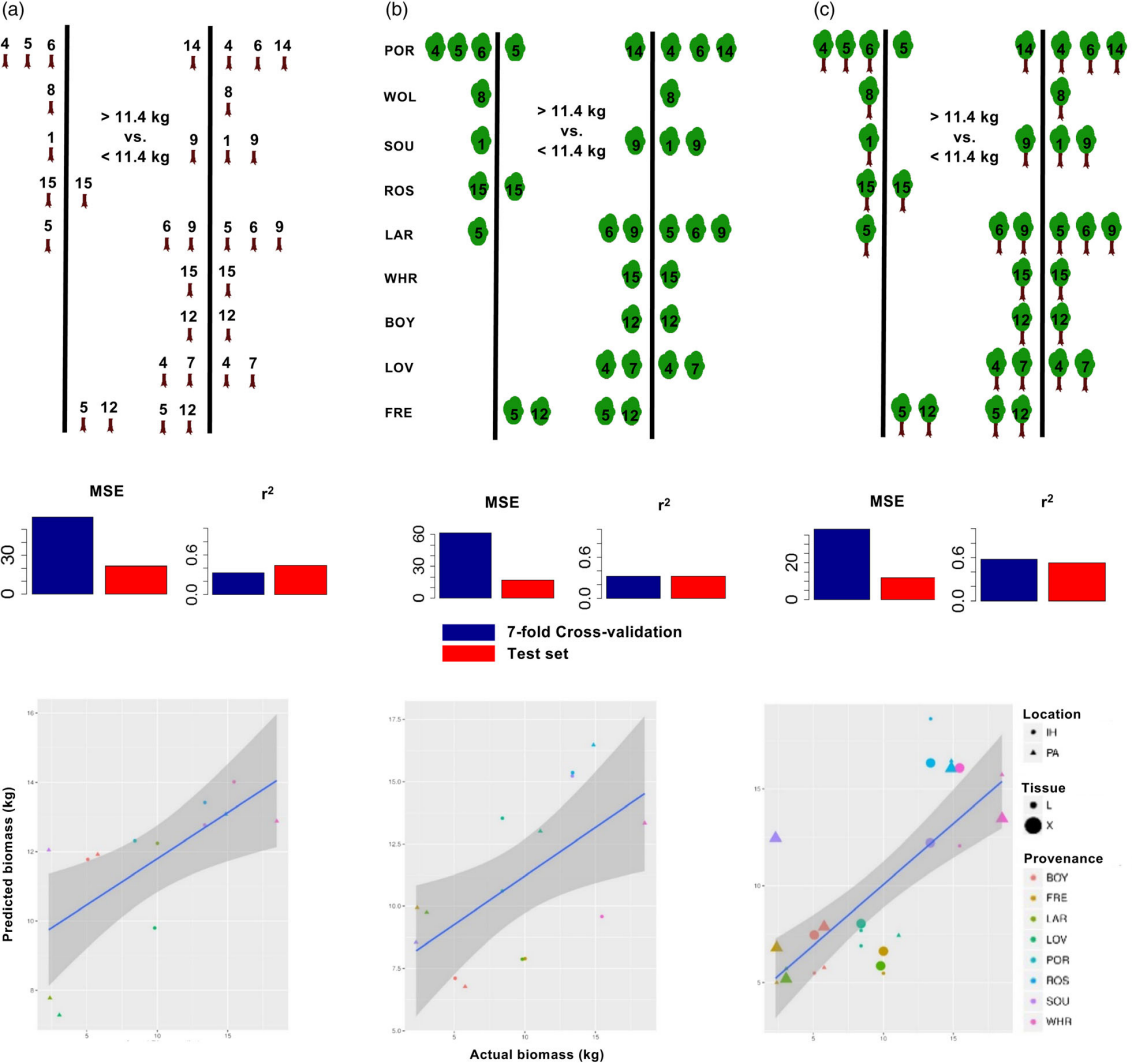
Variable selection and deep learning models estimating tree biomass. Top panels illustrate the strategy used to calculate differential methylation on selected methylomes, based on the average biomass of trees in the training set. Middle section of panels summarizes performance of models built with CpG selected by differential methylation. Bottom panels show scatter plots comparing biomass estimations with the actual biomass of trees in the test set. Regression lines of best fit are shown in blue with confidence intervals at *α* = 0.95 shaded in grey. Variable selection, model performance and estimations were computed using (a) xylem‐derived methylomes or (b) leaf‐derived methylomes or (c) methylomes derived from both tissue sources.

To select CpG for modelling tree biomass, a low heritability trait in poplar (McKown et al., 2014; Porth et al., 2013a,2013b), differential methylation was based on comparisons between trees in the training set larger than the mean biomass (11.4 kg) with trees smaller than the mean. In the first comparison, a Welch’s t‐test was used to identify differentially methylated CpG using only xylem‐derived methylomes (Figure 7a—top). Differential methylation tests were similarly conducted using only the leaf‐derived methylomes (Figure 7b—top) or all methylomes in the training data, irrespective of tissue source (Figure 7c—top). A total of 7,772, 7,937 and 8,000 CpG were selected in these comparisons using an FDR cut‐off of ~1e‐5.

With each of the three sets of differentially methylated CpG as input layers, models were trained to estimate tree biomass (Table S2c). Cross‐validation predictions of biomass computed using each of the three groups of differentially methylated CpG were compared using the adjusted coefficient of determination (*r* ^2^). The best‐fit model, constructed using a mix of both tissue sources, explained 57.5% of variance in biomass in the cross‐validated training set (Figure 7c—middle). The epigenome models of tree biomass generalized to novel genotypes and biomass estimations made on the test methylomes were visualized as scatter plots (Figure 7—bottom). The mixed‐tissue model showed little evidence of overfitting and explained 52.2% of variance in biomass among novel genotypes of the test set (Figure 7c—bottom, Table S3d).

**Figure 8:**
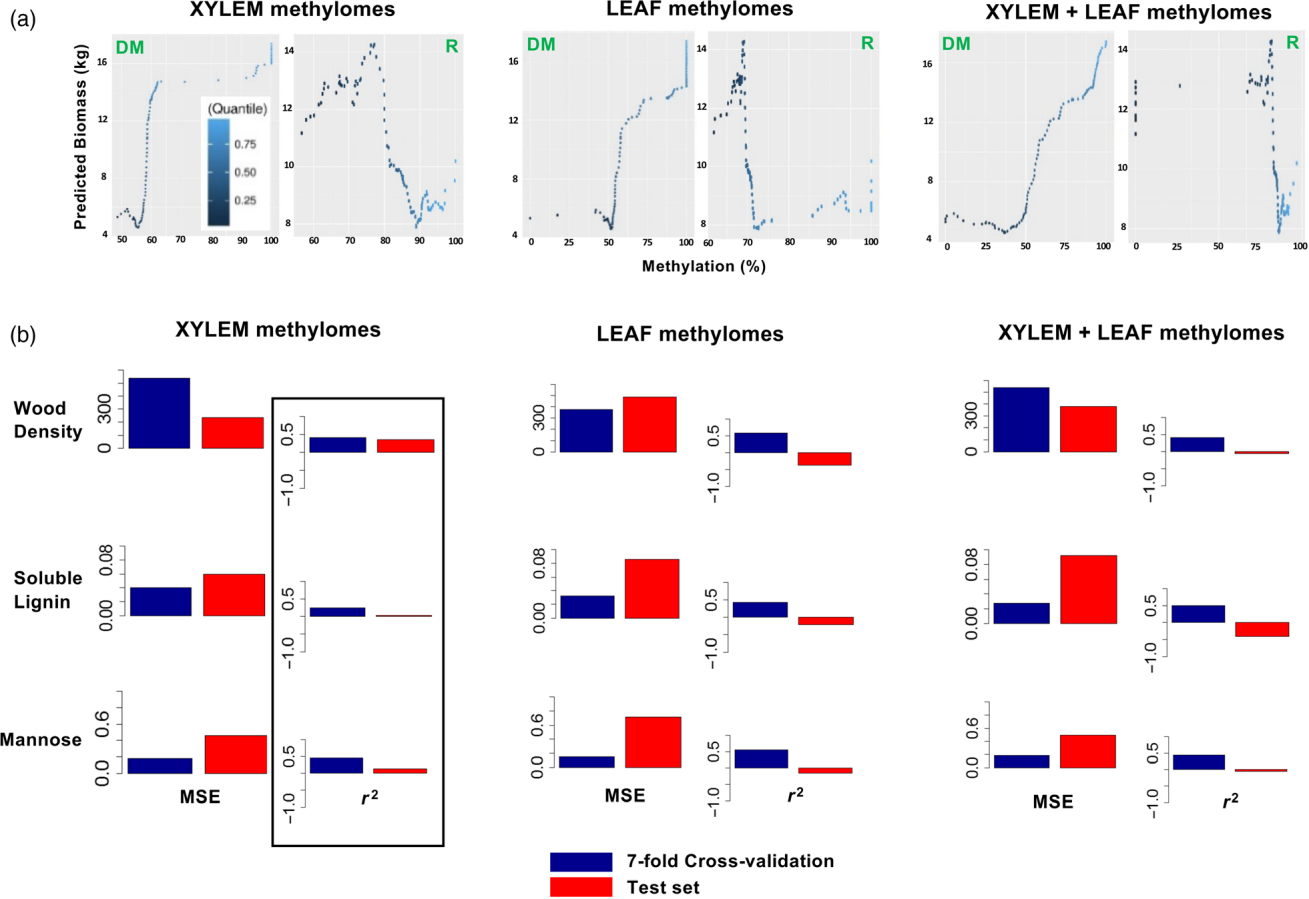
Interpretation of deep learning models estimating quantitative traits. (a) Methylation‐response plots relating levels of input CpG methylation with output biomass estimations. Plots calculated on the basis of xylem‐specific methylation (left), leaf‐specific methylation (middle) or a mix of tissue types (right) are shown. Plots were generated by permuting training data selected by differential methylation (‘DM’) or through random selections of CpG (‘R’). (b) Tissue specificity of models estimating quantitative wood traits. Mean squared error (MSE) and coefficient of determination (*r* ^2^) are shown for cross‐validation and test set estimations obtained using xylem‐specific methylomes, leaf‐specific methylomes or a combination of both tissue types.

Large differences in DNA methylation distinguish poplar xylem and leaves (Kocsis *et al.*, 2003) (Figure 3b); therefore, a visual representation was used to examine how methylomes derived from different tissue sources participate in models estimating biomass. Using the best performing biomass models, methylation‐response plots were generated from 235 million permutations of the training data selected by differential methylation (‘DM’ in Figure 8a). As comparators, biomass estimation using nine randomly chosen selections of 8000 CpG was attempted (three for each tissue split).

Biomass models trained with randomly chosen CpG were strongly overfit to the training data. For example, the best predictions among these models were characterized by an adjusted negative *r* ^2^ of −1.09 on novel genotypes. An additional 235 million permutations of training data were performed, and methylation‐response plots were generated using CpG found in the random models (‘R’ in Figure 8a).

Similar to the previous classification models, patterns relating input methylation with output biomass estimations can be identified using methylation‐response analysis. For CpG selected by differential methylation, model output neurons were activated in a range corresponding to 5–18 kg of biomass. CpG selected randomly activated the output neurons in a range corresponding to 8–15 kg of biomass (Figure 8a). For comparison, the actual biomass of trees in the training data ranged from 1.01 to 24.4 kg (Table S1).

In addition, there are clear differences in the overall shape of the biomass methylation‐response curves. Random selections of CpG associated maximum biomass with a narrow peak of methylation, whereas the differentially methylated CpG assumed a sigmoidal, nonlinear relationship associating increased levels of methylation with increased biomass. For CpG selected by differential methylation, maximum biomass was always associated with the highest level of methylation (Figure 8a). These analyses, along with examination of the variable importance, found no evidence that methylation differences due to tissue type were confounding factors. However, tissue‐specific methylation differences were also apparently not used as features to predict biomass.

### Xylem‐dependent epigenomic estimations of poplar wood properties

Models estimating wood density, soluble lignin content and mannose content were trained and evaluated according to the same procedure used for the biomass models. For each wood trait, differential methylation was used to compare trees with values larger than the mean with those smaller than the mean value. Tissue specificity of these models was evaluated by selecting 8000 ± 100 CpG methylation intensities from xylem comparisons, leaf comparisons or mixed‐tissue comparisons, using an FDR cut‐off of approximately 2e‐4. As was done for the biomass models, hyperparameter searches were conducted using the selections of differentially methylated CpG as input layers and tissue‐specific models were evaluated based on their cross‐validation predictions (Table S2c).

Large proportions of phenotypic variance in wood quality traits (density: 40.9%; soluble lignin content: 25.3%; mannose content: 44.8%) in the cross‐validated training set were explained from natural variation in xylem CpG methylation (box in Figure 8b). The wood trait models showed evidence of overfitting due to leaf‐specific methylation. For each wood trait, estimations did not generalize to novel genotypes in the models trained with leaf‐derived methylomes or a combination of the two tissue types (unboxed analyses in Figure 8b). In the best performing xylem models, 35.0% of variance in wood density, 6.0% of variance in soluble lignin and 13.7% of variance in mannose content were explained among trees in the test set (Figure 8b).

### Robustness of epigenomic estimation to alternate data splits

Given that the number of training and testing examples is small, we considered that performance of one or more of the trait estimations may be inflated due to methylation patterns unique to the constant training/test division of the data (Split 1). To investigate this, the poplar genotypes were separated into two distinct splits relative to Split 1 (Figure S5a). Variable selection by differential methylation was conducted *de novo* using the training methylomes defining Splits 2 and 3 to select 8000 ± 100 CpG (or 600 CpG for provenance) for each split and each trait. Deep learning models were trained using the hyperparameter tuning strategy and selected according to their performance on the cross‐validated training data for each split.

Modelling results using the alternative splits were very similar to results obtained for Split 1 with few exceptions (Figure S5b,c). A reduction in performance was observed for models classifying the provenance of trees in the Split 2 and 3 test sets. This was expected because the variable selection used in provenance models makes use of hand‐crafted features that are likely attuned to Split 1. Tissue dependencies in the quantitative trait models were also observed using the alternative splits. Although tree biomass of novel genotypes was estimated to some degree using any combination of xylem or leaf methylomes, leaf‐derived methylomes confounded the estimation of wood quality traits in the test set. Estimation of soluble lignin content using xylem‐derived methylomes showed a marked increase in performance in Split 3, with 44% and 27% of phenotypic variance explained in the cross‐validated training data and test data, respectively.

Selecting CpG predictors according to differential methylation may not be equally effective for all traits. For example, we were unable to select wood trait‐related CpG at the same level of statistical significance as the biomass‐associated CpG. There are also large differences in variance among the phenotypes. Although 20‐fold differences in biomass exist among the poplar trees, phenotypic variance in soluble lignin content is, in comparison, much lower (Figure 1b). Variable selection techniques and different neural network architectures can be optimized in a larger study containing an independent validation set, and new methods will be required to develop a more automated variable selection strategy for multiclass classifications.

### Natural variation in DNA methylation is an effective trait estimator

Methylomes obtained from different tissue sources were used to examine tissue dependence of the quantitative trait models, and the tree biomass estimations are interesting in several respects. For example, methylomes derived from two tissues were used to model biomass without labelling their tissue source. It is therefore worth investigating whether models constructed by incorporating additional tissues might be more accurate. Secondly, leaf methylomes (obtained from coppiced trees in 2014) were found to be as effective as xylem methylomes (obtained from uncoppiced trees in 2011) in estimating tree biomass in 2011. Given that these leaves could not have causally influenced trees coppiced in 2011, we find the potential to make future predictions of agricultural performance using epigenetic variation very significant. In a manner similar to genomic prediction based on seed genetic variation (Yu *et al.*, 2016), it may be possible to use DNA methylomes obtained from plant seeds or seedlings to predict eventual plant biomass.

To decipher predictive features in patterns of natural epigenetic variation, we developed a visual interpretation of the DNA methylation models. Our method is reminiscent of image analysis methods in which deep learning models are queried to determine which features the layers find important in an image (LeCun *et al.*, 2015; Krishevsky et al., 2012). Deep neural networks in this study solved classification problems by identifying intervals or levels of methylation that distinguish each class in a strictly defined sequence specified by the model. Using similar visualizations, models predictive of tree biomass were characterized by a nonlinear relationship between increasing CpG methylation and increasing biomass.

Controlled genotype and environment designs were previously used to model heritable traits in near‐isogenic *Arabidopsis* lines based on induced differences in DNA methylation (Cortijo *et al.*, 2014; Hu *et al.*, 2015; Kooke *et al.*, 2015). In order to be industrially applicable, we sought to determine whether natural genetic or environmental variation would confound DNA methylation‐based models. Poplar trait estimation in field conditions was attempted and uncovered little evidence that these sources of epigenetic variation were confounding effects on the methylation models. Notably, the ROS ‘provenance’ is unique in several respects, including its exotic genotype. This *P. balsamifera* × *P. deltoides* hybrid included by a sizeable margin the largest trees in our study, and the ROS‐derived methylomes participated in each of the models.

Using epigenomic estimation enabled by deep learning, significant proportions of phenotypic variance in poplar biomass and physico‐chemical wood traits are explained by epigenetic variation. In addition, tissue type and a component of population structure (tree provenance) are classified with high probability. This study thus provides evidence that diverse phenotypes, including weakly heritable or nonheritable traits, can be modelled from natural patterns of CpG methylation.

Predictive CpG, representing less than 0.01% of the total number of cytosines covered by sequencing, was identified using variable selection techniques. The most complex models in this study used 8400 CpG as input, and the smallest model required only 14. For comparison, a model constructed using penalized logistic regression to classify the gender of poplar trees in this study required approximately 20 million cytosines as input (Bräutigam *et al.*, 2017). Trait estimations made on the basis of small amounts of DNA methylation data have potential in authenticating the identity, provenance or quality of agroforestry products using technologies similar to genomic prediction. For example, pretrained machine learning models could compute hundreds of economically useful estimations from a DNA sample spotted on a methylation microarray.

## Experimental procedures

### Growth and phenotyping of *Populus balsamifera*

Genetic material used in this study is a subset of a larger Agriculture Canada Balsam Poplar (AgCanBaP) collection representing 65 provenances composed of 15 distinct trees sampled per provenance. Details regarding the origin and sampling procedure used to establish the 9 provenances used in this study are previously described (Soolanayakanahally et al., 2009). The provenances employed were POR—Portage, WOL—Wollaston Lake, SOU—Sioux Lookout, ROS—Roseville, LAR—La Ronge, WHR—Whitehorse, BOY—Boyle, LOV—Love and FRE—Fredericton.

From each tree, 6‐ to 9‐cm dormant whips with a minimum of two buds were forced to root in Spencer‐Lemaire rootrainers filled with a mixture of Sunshine‐2 (Sun Gro Horticulture, Vancouver, Canada) growing mix (60%), peat moss (30%) and vermiculite (10%). The rooted cuttings were grown in a greenhouse during the spring of 2006 with natural light supplemented with artificial lighting by cool‐white fluorescent lamps to provide a 21‐h photoperiod and a minimum fluence of 400 μmol m^‐2^ s^‐1^ PPFD at plant level. Maximum day and night temperatures were maintained close to 25 and 18°C, respectively. Upon flushing, the plants were well watered and fertilized with Hoagland’s solution. In early June, when all plants were approximately 30 centimetres tall, they were moved to a shade house.

Common gardens were established at Prince Albert (53.62°N 106.43°W; elevation 461 m) and Indian Head (50.52°N 103.68°W; elevation 605 m), Saskatchewan, in May 2007. Fifteen genotypes from each provenance were planted in a group at 2m × 2m spacing, and groups (provenances) were then randomized within blocks (i.e. five ramets per genotype). Usual growing season length in Indian Head is longer than Prince Albert by approximately 17 days. Maximal photoperiods for Prince Albert and Indian Head are 16h 51 min and 16 h 12 min, respectively.

Trees were harvested at both common garden sites for biomass as well as physical and chemical wood quality measurements in October 2011. In early July 2014, the third fully expanded leaf from growing tips of coppiced tissue was collected from the same balsam poplar genotypes. Both leaf and xylem samples were flash‐frozen in liquid N_2_, transported to the University of Toronto, Scarborough, and stored at −80°C until further use.

### Whole‐genome bisulphite sequencing of *Populus balsamifera* DNA

Frozen tissue samples were ground to a fine powder in liquid N_2_ using prechilled mortar and pestles. DNA was extracted from 100 mg of each powdered sample using the CTAB method (Murray and Thompson, 1980) with slight modification: To the CTAB extraction buffer was added 200 units/mL of RNAse A and 0.4 g/mL PVP (MW 40000) (Sigma‐Aldrich, Oakville, ON, Canada). Extracted DNA was precipitated twice with isopropanol and once with 75% EtOH and resuspended in 50–100 µL of 0.1X TE buffer supplemented with 0.2 units of RNAse A. DNA integrity was assessed using agarose gel electrophoresis and quantitated with a Quant‐iT PicoGreen dsDNA Assay Kit (Thermo Fisher Scientific, Mississauga, ON, Canada) on a Bio‐Tek Powerwave HT Microplate Reader.

Purified DNA samples were submitted for sodium bisulphite treatment and sequencing at the Michael Smith Genome Sciences Centre (Vancouver, Canada) according to established procedure (Lister *et al.*, 2008). Sequencing libraries were constructed using a TruSeq DNA HT (Illumina, Vancouver, BC, Canada) Sample Kit, the original Illumina adaptors, and with a target insert sequence of 300 nucleotides. 10 ng of lambda DNA was spiked into each sequencing sample for later bisulphite conversion analysis. DNA was sequenced, 2–3 samples per lane, at a target read length of 125 cycles/end on an Illumina HiSeq 2500 instrument.

### Bioinformatics

All computations were performed using a Dell Precision T7610 Workstation with 64 GB RAM and two Intel Xeon E5‐2600 processors with 12 cores each, and running Ubuntu Server 12.04.5.

Following an initial filter selecting for Illumina chastity‐passed sequences, adaptors and ambiguous bases were removed from reads with Trimmomatic (Bolger *et al.*, 2014) v0.30 in paired‐end mode using the ILLUMINACLIP algorithm. Unpaired reads, as well as those of length < 36 nt, were excluded from all further analyses. Paired reads passing these criteria were aligned against the Ptrichocarpa_210_v3.0 reference genome using Bismark (Krueger & Andrews 2011) v0. 12.5 in directional mode with a score_min L,0,‐0.6. Bisulphite conversion was estimated following alignment of paired reads against a lambda reference genome (GenBank J02459.1) using Bismark in directional mode with a stringent score_min L,0,‐0.2. The frequency of methylated cytosines present in different contexts (CpG, CHG and CHH) was estimated using the bismark_methylation_extractor function in Bismark. Chromosomal positions of CpG reported in this study are therefore relative to the *P. trichocarpa* v3 reference genome.

Analyses of neural networks constructed in this study make use of a variable importance calculation designed to rank or score the relative contribution of cytosines to the phenotypic estimations. The potential influence of methylation context or strand‐specific methylation on variable importance estimation is unknown. In order to make each cytosine in the DNA methylation models as comparable as possible, neural networks were constructed using only the strand‐symmetric CpG.

Methylation intensity (0%–100%) of individual CpG in each sequenced sample was measured as the proportion of conversion using the read.bismark function in the methylKit v.0.9.5 R package. Within a sequenced sample, CpG covered by fewer than 10 reads were excluded as a prefilter. The complete data set used for modelling consisted of 1.3 billion CpG methylation intensities (12 358 704 CpG/methylome × 105 methylomes), of which 40 227 581 (3.1 % of the total) were not obtained in one or more methylomes. These missing data were treated according to an impute‐as‐needed approach in subsequent analyses. PCA, t‐SNE, HCA and modelling by deep learning require no missing data so it was imputed as the mean methylation level of cytosines among methylomes at that position. Missing values were imputed into the training and test data using separate calculations to preserve the independence of the two groups.

Annotation of CpG with respect to *P. trichocarpa* gene models was carried out using the annotate.WithGenicParts function in the methylKit package. *P. trichocarpa* gene models were mapped to their best BLAST result in the TAIR10 *A. thaliana* annotation (Porth et al., 2013a,2013b).

### Statistical analyses

Box plots were produced using the multcompView v.0.1‐7 R package. Significant differences among groups were calculated *post hoc* according to Tukey’s HSD test. Hierarchical clustering of methylation data was conducted with pvclust v2.0.0 and pheatmap v1.0.8 R packages using the Ward clustering method computed on Euclidean distances. PCA was conducted using the prcomp R package without centring or scaling, and results were plotted using the fviz_pca_var function in the factoextra v1.0.3 R package. T‐statistic stochastic neighbour embedding (t‐SNE) of methylation data was conducted using the Rtsne v0.10 R package following initial PCA dimensionality reduction to 100 dimensions, perplexity ranging from 5 to 23, theta = 0.1 and 20000 iterations. t‐SNE results were plotted in three dimensions using the scatterplot3d v0.3‐37 R package. Differential methylation of individual CpG was calculated by Welch’s t‐test using the mt.teststat function in the multtest v2.24.0 R package at a level of significance *α* = 0.01. Correction of *P*‐values after multiple testing was carried out according to the FDR method (Hochberg and Benjamini, 1990).

### Deep learning models based on CpG methylation

Associations between CpG methylation and poplar traits were modelled by constructing supervised feed‐forward artificial neural networks using the R interface and deep learning functions in the h2o.ai v3.2.0.3 software package (://h2orelease.s3.amazonaws.com/h2o/latest_stable_Rdoc.html). All models were trained using an input layer composed of individual CpG methylation intensities and two fully connected hidden layers. Output layers consisted of multiple neurons corresponding to predicted classes implemented with the softmax function or a single output neuron for regression predictions. Models were trained to minimize a specified loss function using a parallelized method of stochastic gradient descent (Recht *et al.*, 2011) computed by backpropagation (LeCun et al., 1998). The number of epochs used for training varied from 200 to 600.

Model hyperparameters were assayed using grid searches: (i) the number of neurons in each hidden layer ranged from 4 to 400, (ii) the activation function used at each hidden layer was Tanh, Rectifier or Maxout, (iii) the value of the L_1_ (Lasso) regularizer ranged from 5e‐6 to 5e‐4, and (iv) the value of the L_2_ (Ridge) regularizer ranged from 5e‐7 to 5e‐5. The proportion of neurons randomly dropped from the input layer and hidden layers with each training epoch ranged from 0% to 50%. Remaining parameters were set at default values in the h20.ai package.

Performance of each model was estimated following sevenfold cross‐validation of the data used for model training. Models were built using 86% of the training data, while 14% of data were held out for each of the 7 models. Holdout predictions were then scored against their true labels, and overall cross‐validation metrics were computed.

Variable importance is an active topic of investigation, and there are several methods for analysing this property of input variables in artificial neural networks (Garson, 1991; Gedeon, 1997; Ibrahim, 2013). All models constructed in this study employ two hidden layers of neurons. For this reason, we used an algorithm designed to calculate the influence of weights connecting input features to two hidden layers (Gedeon, 1997) in order to score/rank the relative variable importance.

### Interpretation of deep learning models

Weights and biases were extracted from a fit model using the export_weights_and_biases parameter in the h2o.deeplearning function. Variable importance, weights and biases were ordered using custom R scripts and used as input to construct neural interpretation diagrams using the plotnet function in the NeuralNetTools v1.4.0 R package.

Relationships between values of input variables and output neuron activation were examined using a variation of the ‘Profile’ method of sensitivity analysis (Gevrey *et al.*, 2003; Lek *et al.*, 1996). Data used to plot methylation‐response curves for each CpG in a deep learning model were obtained in two steps. In the first step, CpG methylation values were simulated from the data used during model training. For each CpG, methylation values were calculated at 99 quantiles (ranging from 0.01 to 0.99 in increments of 0.01) with respect to its methylation in the training set. For each methylation value of an individual CpG, values of the remaining CpG in the model were permuted as 99 quantiles (ranging from 0.01 to 0.99 in increments of 0.01) of their values in the training data. In the second step, these permuted methylation data (99 × 99 = 9801 permutations per cytosine) were used as input to a fit model where activation of the output neuron(s) was recorded. Using the ggplot2 v2.1.0 package in R, the activation of each output neuron was plotted on scatter plots as a function of methylation of each input CpG.

## Conflict of interest

The authors declare no conflicts of interest related to this work.

## Author contribution

MMC conceived the study. MJC, SDM, RYS and MMC designed the experiment. FU, OS, RYS and SDM contributed to fieldwork and phenotypic measurements. MJC performed molecular and computational analyses. MJC, SDM, RYS and MMC prepared and revised the manuscript.

## Supporting information


**Figure S1** WGBS analysis of cytosine methylation in 105 *P. balsamifera *DNA samples.

**Figure S2** Additional methylation‐response plots.

**Figure S3** Confusion matrices summarizing provenance predictions made by different models.

**Figure S4** Methylation levels among 14 tissue‐predictive CpG.

**Figure S5** Robustness of the epigenomic estimation method to alternate splits of the data.

**Table S1** Phenotypes of 50 *P. balsamifera *trees grown at either Prince Albert or Indian Head, Saskatchewan, Canada.

**Table S2** Deep learning parameter settings used to train models.

**Table S3** Summary of predictions made by deep learning models.
