# Supplementary material for: Learning from methylomes: epigenomic correlates of Populus balsamifera traits based on deep learning models of natural DNA methylation
Source: Plant Biotechnol J. 2019 Dec 18;18(6):1361–75. doi: 10.1111/pbi.13299 (PMC7207000; doi:10.1111/pbi.13299)
Supplement: Supplementary file 1 — Figure S1 WGBS analysis of cytosine methylation in 105 P. balsamifera DNA samples. [file PBI-18-1361-s001.pdf]

Supplementary Figure 1

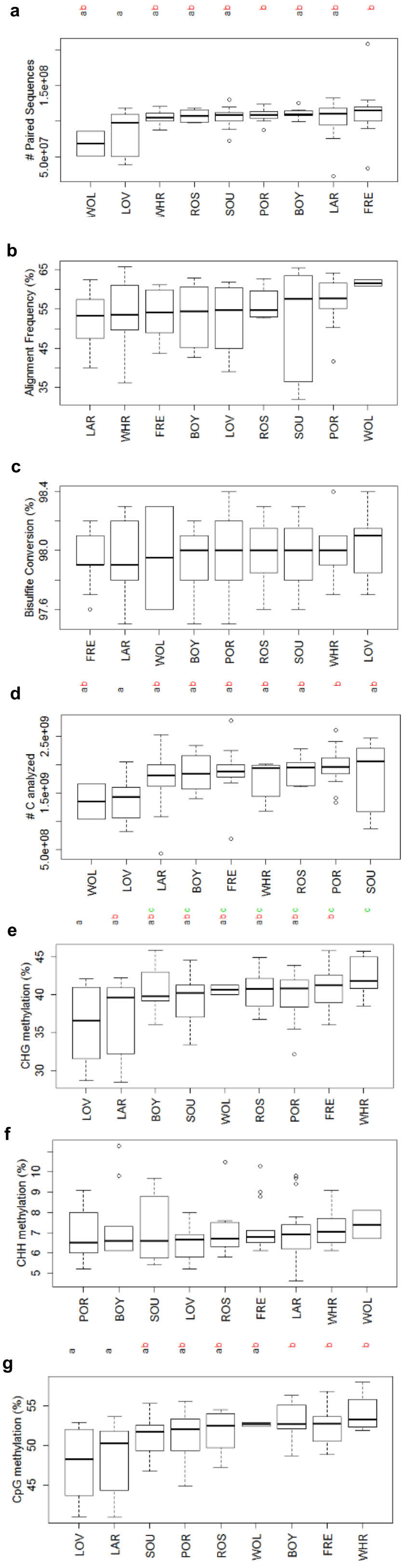

Figure S1. WGBS analysis of cytosine methylation in 105 *P. balsamifera* DNA samples. Whole-genome bisulfite sequencing was conducted on 50 xylem and 55 leaf DNA samples isolated from 9 provenances of *P. balsamifera* growing in two common gardens. Bioinformatics analyses are summarized as boxplots grouping samples by provenance. Letters indicate significant differences among groups according to Tukey's HSD test. (a) Number of sequence pairs passing quality control measures and used in alignments. (b) Frequency (%) of paired *P. balsamifera* – derived sequences aligned to the *P. trichocarpa* v3 reference genome. (c) Estimate of bisulfite conversion efficiency based on conversion of spiked-in, unmethylated lambda DNA. (d) Number of cytosines in each sample covered by at least 10 sequence reads. (e,f,g) Frequency (%) of cytosine methylation in CHG, CHH and CpG contexts, respectively.
