# Supplementary material for: Learning from methylomes: epigenomic correlates of Populus balsamifera traits based on deep learning models of natural DNA methylation
Source: Plant Biotechnol J. 2019 Dec 18;18(6):1361–75. doi: 10.1111/pbi.13299 (PMC7207000; doi:10.1111/pbi.13299)
Supplement: Supplementary file 2 — Figure S2 Additional methylation‐response plots. [file PBI-18-1361-s002.pdf]

Supplementary Figure 2

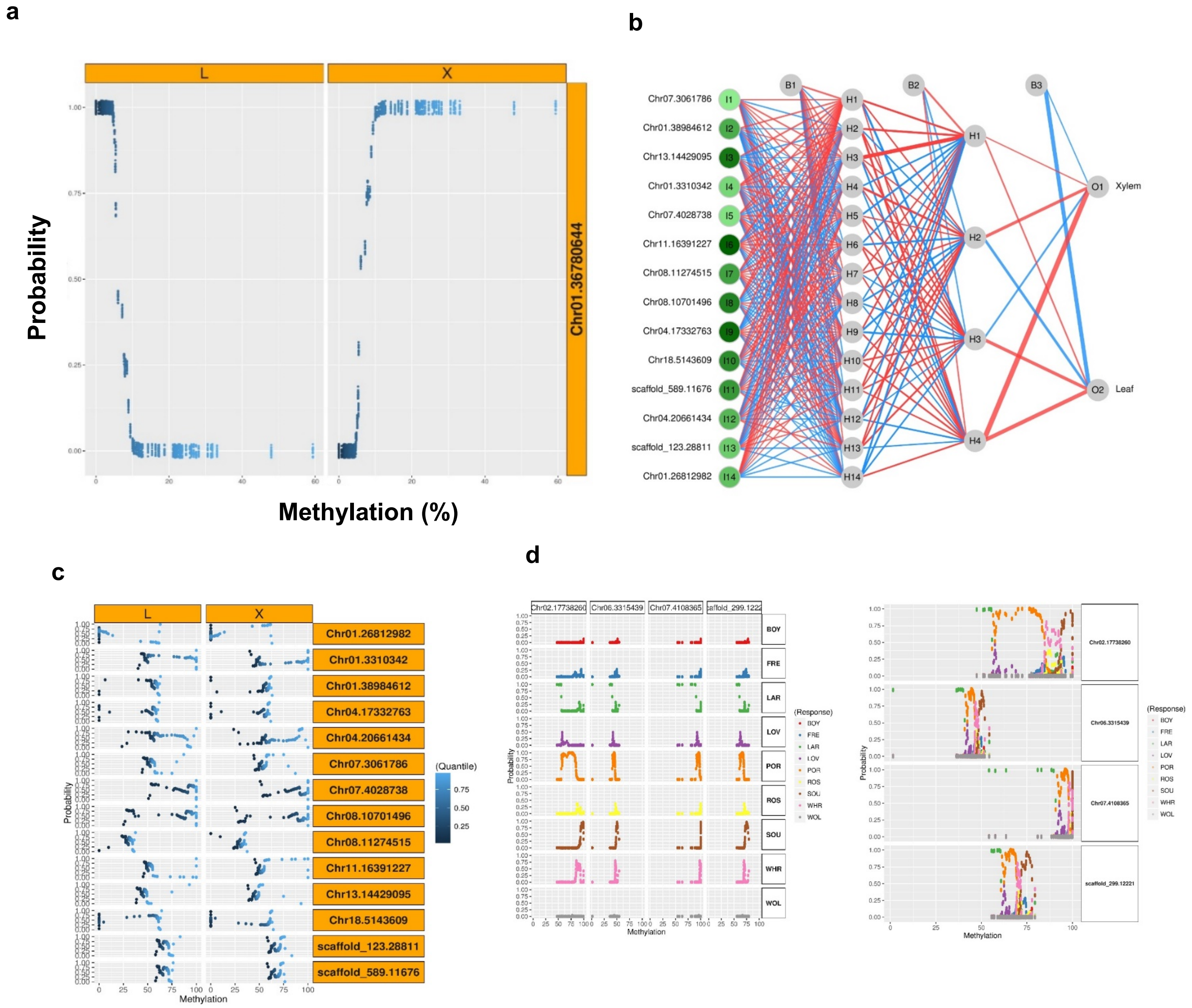

Figure S2. Additional methylation-response plots. (a) Magnification of methylation-response plot produced for Chr01.36780644 in the best-fit 14 CpG tissue classification model (Figure 5c). Distribution of points along the horizontal axis represents 99 quantile methylation values for this cytosine. Vertical "stacks" of points represent tissue probabilities calculated for each of 99 quantile values of the remaining 13 CpG in the model. In the vertical dimension, points are jittered by a factor of 0.3 to highlight small differences between points. (b) Neural interpretation diagram produced for a poorly-fit model constructed with 14 randomly selected CpG. (c) Methylation-response plots produced for the tissue classification model constructed with 14 randomly selected CpG. (d) Methylation-response plots produced for a provenance classification model constructed with 120 randomly selected CpG.
