# Supplementary material for: Learning from methylomes: epigenomic correlates of Populus balsamifera traits based on deep learning models of natural DNA methylation
Source: Plant Biotechnol J. 2019 Dec 18;18(6):1361–75. doi: 10.1111/pbi.13299 (PMC7207000; doi:10.1111/pbi.13299)
Supplement: Supplementary file 3 — Figure S3 Confusion matrices summarizing provenance predictions made by different models. [file PBI-18-1361-s003.pdf]

Supplementary Figure 3

a **Test set, 600 CpG selected by t-SNE**

	BOY	FRE	LAR	LOV	POR	ROS	SOU	WHR	WOL	Err
**BOY**	4	0	0	0	0	0	0	0	0	0/4
**FRE**	0	5	0	0	0	0	0	0	0	0/5
**LAR**	0	0	4	0	0	0	0	0	0	0/4
**LOV**	0	0	0	3	1	0	0	0	0	1/4
**POR**	0	0	1	0	3	0	0	0	0	1/4
**ROS**	0	1	0	0	0	0	3	0	0	4/4
**SOU**	0	0	1	0	0	0	3	0	0	1/4
**WHR**	0	0	0	0	0	0	0	0	4	0/4

b **Full data set, 600 CpG selected by t-SNE**

	BOY	FRE	LAR	LOV	POR	ROS	SOU	WHR	WOL	Err
**BOY**	9	0	0	0	0	0	0	0	0	0/8
**FRE**	0	14	0	0	0	0	0	0	0	0/14
**LAR**	0	0	16	0	0	0	0	0	0	0/16
**LOV**	0	0	0	12	0	0	0	0	0	0/12
**POR**	0	0	0	0	19	0	0	0	0	0/19
**ROS**	0	0	0	0	0	8	0	0	0	0/8
**SOU**	0	0	0	0	0	0	12	0	0	0/12
**WHR**	0	0	0	0	0	0	0	8	0	0/8
**WOL**	0	0	0	0	0	0	0	0	4	0/4

c **Full data set, 120 CpG selected randomly**

	BOY	FRE	LAR	LOV	POR	ROS	SOU	WHR	WOL	Err
**BOY**	4	0	2	1	0	0	2	0	0	4/8
**FRE**	0	11	1	0	1	0	0	0	1	3/14
**LAR**	1	2	9	1	1	0	1	1	0	7/16
**LOV**	0	1	4	5	1	0	1	0	0	7/12
**POR**	0	0	0	1	15	0	1	1	1	4/19
**ROS**	0	1	1	0	0	4	2	0	0	4/8
**SOU**	0	0	3	0	0	1	7	0	1	5/12
**WHR**	0	0	0	0	2	0	0	6	0	2/8
**WOL**	0	1	0	0	1	0	1	0	1	3/4

Figure S3. Confusion matrices summarizing provenance predictions made by different models. Variables used for model training and methylomes used as the prediction set are indicated.
