# Supplementary material for: Learning from methylomes: epigenomic correlates of Populus balsamifera traits based on deep learning models of natural DNA methylation
Source: Plant Biotechnol J. 2019 Dec 18;18(6):1361–75. doi: 10.1111/pbi.13299 (PMC7207000; doi:10.1111/pbi.13299)
Supplement: Supplementary file 4 — Figure S4 Methylation levels among 14 tissue‐predictive CpG. [file PBI-18-1361-s004.pdf]

Supplementary Figure 4

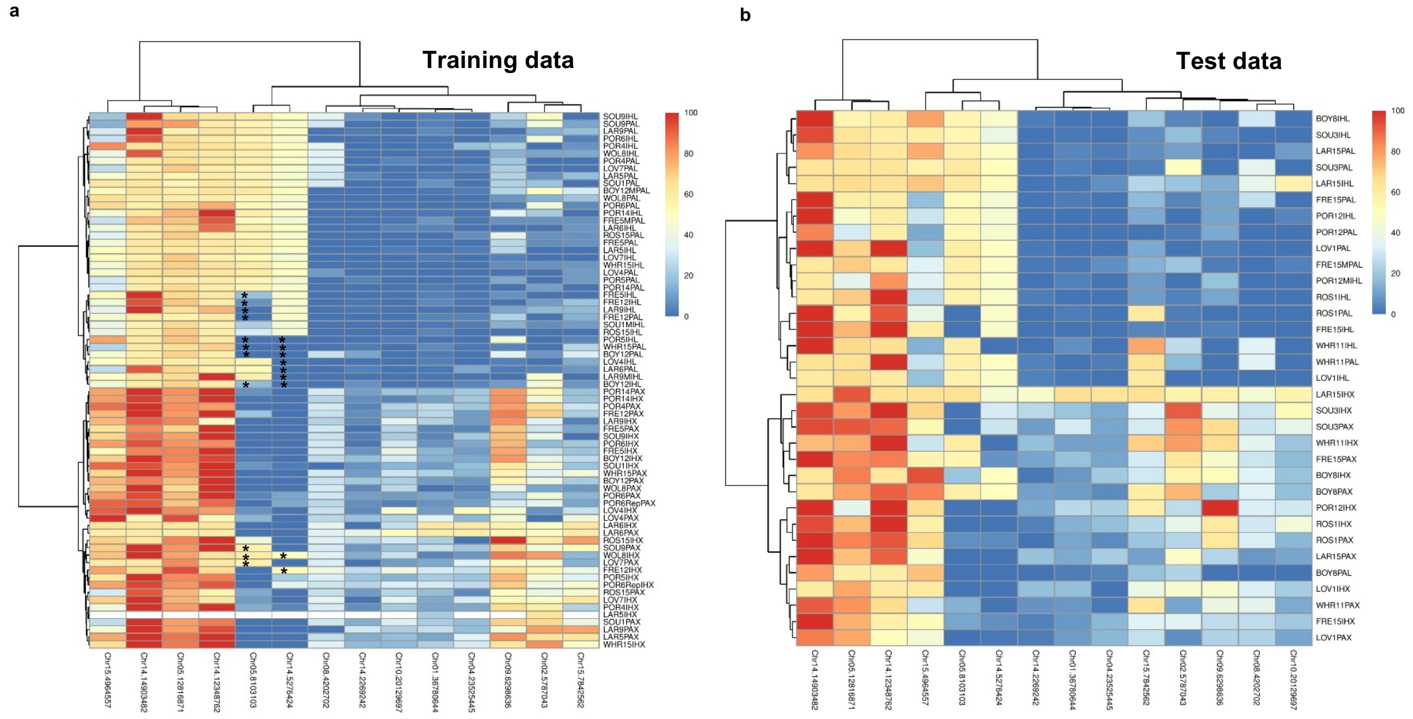

Figure S4. Methylation levels among 14 tissue-predictive CpG. Hierarchical clustering and heatmap of (a) 72 training samples, and (b) 33 test samples, based on methylation of 14 tissue-predictive CpG. Chromosomal locations of CpG with respect to the *P. trichocarpa* v3 genome reference is indicated at bottom. Asterisks denote atypical features of the training data associated with tissue classification.
