# Supplementary material for: Learning from methylomes: epigenomic correlates of Populus balsamifera traits based on deep learning models of natural DNA methylation
Source: Plant Biotechnol J. 2019 Dec 18;18(6):1361–75. doi: 10.1111/pbi.13299 (PMC7207000; doi:10.1111/pbi.13299)
Supplement: Supplementary file 5 — Figure S5 Robustness of the epigenomic estimation method to alternate splits of the data. [file PBI-18-1361-s005.pdf]

a

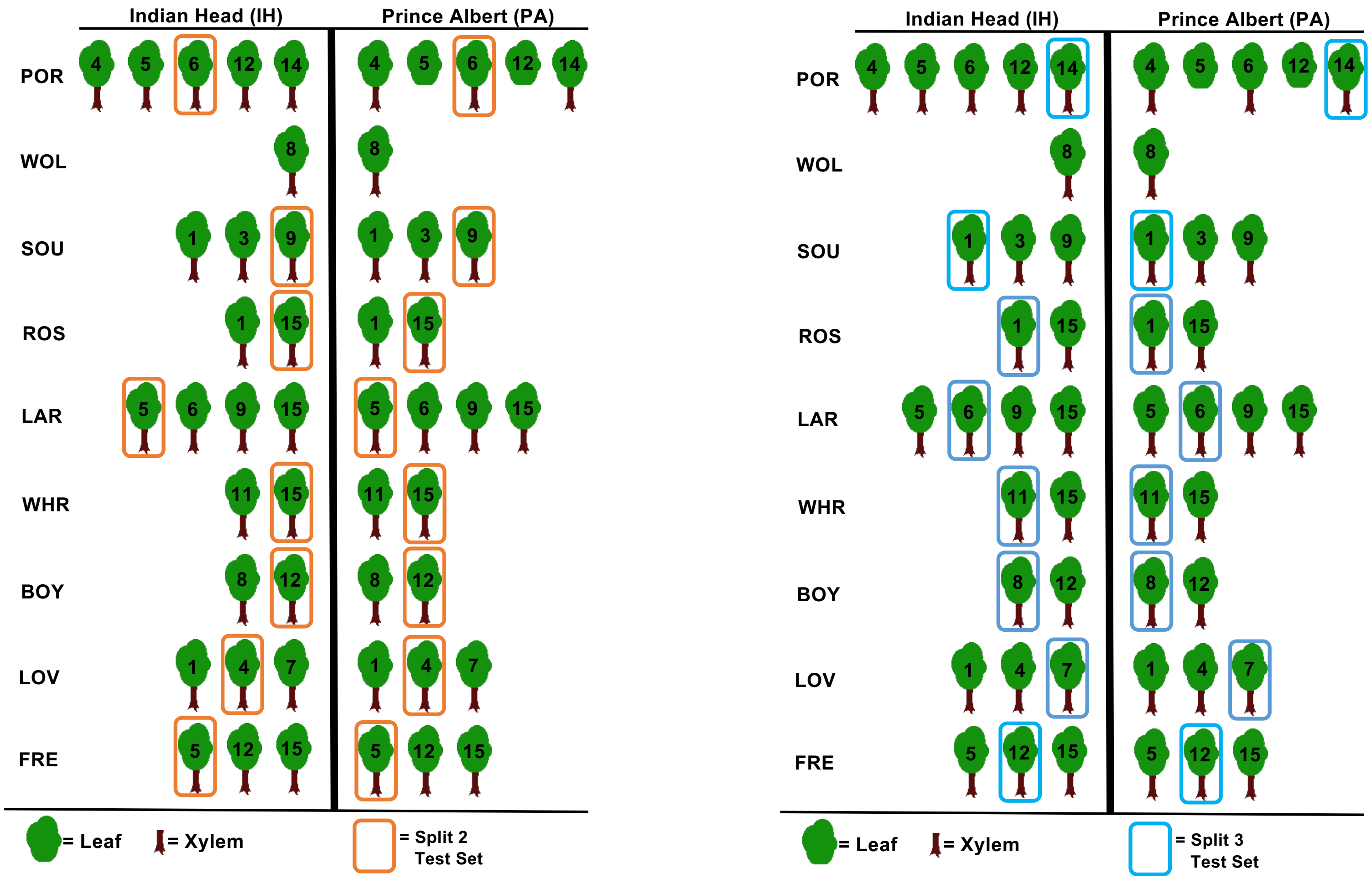

Figure S5. Robustness of the epigenomic estimation method to alternate splits of the data. (a) Schematic representation of Split 2 and Split 3, in which different *P. balsamifera* genotypes are used as the independent test set. (b) Tabulated results of classification models computed using the variable selection strategy used for Split 1. (c) Tabulated results of regression models computed using the variable selection strategy used for Split 1.

b

Phenotype	7-Fold Cross-Validation		Test Set	
	Log-loss	Error	Log-loss	Error
Tissue (Split 2)	3.5e-15	0/64	3.3e-11	0/41
Tissue (Split 3)	6.8e-18	0/73	6.3e-12	0/32
Accession (Split 2)	0.13	3/64	3.14	14/41
Accession (Split 3)	0.19	4/73	3.67	12/32

c

Phenotype	Xylem + Leaf				Xylem only				Leaf only			
	7-Fold CV		Test Set		7-Fold CV		Test Set		7-Fold CV		Test Set	
	MSE	r^2	MSE	r^2	MSE	r^2	MSE	r^2	MSE	r^2	MSE	r^2
Biomass (Spl 2)	7.64	0.69	73.6	0.40	7.44	0.69	109	0.19	7.88	0.56	105	0.29
Biomass (Spl 3)	22.4	0.55	23.0	0.36	36.5	0.59	34.2	0.15	51.9	0.41	23.1	0.36
Density (Spl 2)	266	0.60	896	-0.06	307	0.21	881	-0.02	216	0.41	848	0.01
Density (Spl 3)	294	0.53	966	-0.19	140	0.76	792	0.02	188	0.63	998	-0.23
Sol. Lignin (Spl 2)	0.04	0.02	0.09	-0.16	0.02	0.56	0.07	0.03	0.03	0.17	0.10	-0.20
Sol. Lignin (Spl 3)	0.03	0.41	0.09	-0.35	0.03	0.44	0.05	0.27	0.03	0.44	0.38	-0.56
Mannose (Spl 2)	0.19	0.19	0.24	-0.51	0.18	0.56	0.13	0.18	0.18	0.62	0.24	-0.55
Mannose (Spl 3)	0.14	0.14	0.81	-0.16	0.09	0.50	0.68	0.27	0.12	0.56	0.79	-0.15
